# Construction of an M2 macrophage-related prognostic model in hepatocellular carcinoma

**DOI:** 10.3389/fonc.2023.1170775

**Published:** 2023-06-20

**Authors:** Huangqin Song, Xiaoxiao Wang, Chao Zhang, Jiefeng He

**Affiliations:** ^1^ Third Hospital of Shanxi Medical University, Shanxi Bethune Hospital, Shanxi Academy of Medical Sciences, Tongji Shanxi Hospital, Taiyuan, China; ^2^ Shanxi Bethune Hospital, Shanxi Academy of Medical Sciences, Tongji Shanxi Hospital, Third Hospital of Shanxi Medical University, Taiyuan, China; ^3^ Tongji Hospital, Tongji Medical College, Huazhong University of Science and Technology, Wuhan, China

**Keywords:** hepatocellular carcinoma, M2 macrophages, prognostic model, tumor microenvironment, LASSO

## Abstract

**Background:**

M2 macrophages play a crucial role in promoting tumor angiogenesis and proliferation, as well as contributing to chemotherapy resistance and metastasis. However, their specific role in the tumor progression of hepatocellular carcinoma (HCC) and their impact on the clinical prognosis remain to be further elucidated.

**Materials and methods:**

M2 macrophage-related genes were screened using CIBERSORT and weighted gene co-expression network analysis (WGCNA), while subtype identification was performed using unsupervised clustering. Prognostic models were constructed using univariate analysis/least absolute shrinkage selector operator (LASSO) Cox regression. In addition, Gene Ontology (GO)/Kyoto Encyclopedia of Genes and Genomes (KEGG), gene set enrichment analysis (GSEA), gene set variation analysis (GSVA), and mutation analysis were used for further analysis. The relationship between the risk score and tumor mutation burden (TMB), microsatellite instability (MSI), the efficacy of transcatheter arterial chemoembolization (TACE), immunotype, and the molecular subtypes were also investigated. Moreover, the potential role of the risk score was explored using the ESTIMATE and TIDE (tumor immune dysfunction and exclusion) algorithms and stemness indices, such as the mRNA expression-based stemness index (mRNAsi) and the DNA methylation-based index (mDNAsi). In addition, the R package “pRRophetic” was used to examine the correlation between the risk score and the chemotherapeutic response. Finally, the role of *TMCC1* in HepG2 cells was investigated using various techniques, including Western blotting, RT-PCR and Transwell and wound healing assays.

**Results:**

This study identified 158 M2 macrophage-related genes enriched in small molecule catabolic processes and fatty acid metabolic processes in HCC. Two M2 macrophage-related subtypes were found and a four-gene prognostic model was developed, revealing a positive correlation between the risk score and advanced stage/grade. The high-risk group exhibited higher proliferation and invasion capacity, MSI, and degree of stemness. The risk score was identified as a promising prognostic marker for TACE response, and the high-risk subgroup showed higher sensitivity to chemotherapeutic drugs (e.g., sorafenib, doxorubicin, cisplatin, and mitomycin) and immune checkpoint inhibitor (ICI) treatments. The expression levels of four genes related to the macrophage-related risk score were investigated, with *SLC2A2* and *ECM2* showing low expression and *SLC16A11* and *TMCC1* exhibiting high expression in HCC. *In vitro* experiments showed that *TMCC1* may enhance the migration ability of HepG2 cells by activating the Wnt signaling pathway.

**Conclusion:**

We identified 158 HCC-related M2 macrophage genes and constructed an M2 macrophage-related prognostic model. This study advances the understanding of the role of M2 macrophages in HCC and proposes new prognostic markers and therapeutic targets.

## Background

Hepatocellular carcinoma (HCC) is a dominant subtype of primary liver cancer that has the fastest-growing cancer-related mortality rate globally (1). The 5-year survival rate of HCC remains below 12%, primarily due to the low early detection rates and limited treatment options (2, 3). As a result, identifying robust prognostic markers is crucial to optimizing prognostic accuracy and creating effective risk stratification models, particularly given the survival differences caused by tumor heterogeneity.

Recent cancer research has focused on exploring how the tumor microenvironment (TME) affects tumor progression to achieve therapeutic breakthroughs (4–6). TME–tumor cell interactions play a significant role in modulating the pro-cancer and anticancer roles in biological processes, impacting tumor metabolism and growth and modifying treatment responses (6–8). M2 macrophages comprise the major macrophage subtype in the TME and display tumor-promoting properties (9–11). They contribute to tumor angiogenesis and proliferation, chemotherapy resistance, and metastasis (12, 13). Several studies have confirmed that the degree of macrophage infiltration in the TME correlates positively with poor prognosis in patients with cancer (14–19). However, the role of M2 macrophages in the tumor progression of HCC and their influence on the clinical prognosis remain unclear.

In this research, CIBERSORT and weighted gene co-expression network analysis (WGCNA) were employed using data from The Cancer Genome Atlas Liver Hepatocellular Carcinoma (TCGA-LIHC) to identify M2 macrophage-related genes. The unsupervised clustering approach revealed two M2-related subtypes, which were compared using Gene Ontology (GO) and the Kyoto Encyclopedia of Genes and Genomes (KEGG) to determine their functional differences. A four-gene prognostic model was developed utilizing univariate and least absolute shrinkage selector operator (LASSO) Cox regression analyses, and a nomogram was constructed to predict HCC prognosis. In addition, gene set variation analysis (GSVA), gene set enrichment analysis (GSEA), mutational analysis, and TME analysis were performed to identify differences between the low and high-risk score groups. This study further explored the relationship between the risk score and microsatellite instability (MSI), transcatheter arterial chemoembolization (TACE) treatment efficacy, tumor purity, TIDE (tumor immune dysfunction and exclusion) score, immunotype, and molecular subtypes, as well as drug sensitivity. The expression and functional roles of the genes linked to the risk score were ultimately validated through experimental verification. Overall, this study effectively developed a four-gene signature associated with M2 macrophages to predict the prognosis of patients with HCC.

## Materials and methods

### Data collection

The gene expression profiles (in fragments per kilobase of transcript per million mapped reads, FPKM) and the clinical information of the LIHC cohort was collected from The Cancer Genome Atlas (TCGA), which included 374 HCC tissues and 50 normal liver tissues. The FPKM values were then transformed into TPM (transcripts per million) samples. As a validation set, the Liver Cancer—RIKEN, JP Project from the International Cancer Genome Consortium (ICGC-LIRI-JP) dataset was used, which included the expression profiles and clinical data of 212 samples from the HCCDB data portal (http://lifeome.net/database/hccdb).

### Identification of M2 macrophage-related genes

CIBERSORT was used to analyze the relative frequencies of 22 immune cell subtypes in 374 HCC samples (18). To assess the correlation among the 22 immune cells, Spearman’s correlation coefficient was utilized. With a soft thresholding of 9, as recommended in the WGCNA guidelines, WGCNA was conducted using the “WGCNA” package, which identified 17 WGCNA modules (20). The MEblack model was chosen as the M2 macrophage-related model through analysis of the module–trait relationship. Protein–protein interaction (PPI) analysis was performed based on the 158 genes in the MEblack model, and the hub genes were identified using the “Degree” method.

### Identification of M2 macrophage-related subtypes

Using the 158 M2 macrophage-related genes in the MEblack model, unsupervised clustering was performed on the 374 HCC samples using the R package “ConsensusClusterPlus.” Based on the cumulative distribution functions (CDFs), the optimal number of clusters (*k*) was determined to be 2 (21). Principal component analysis (PCA) was conducted using the R package “ggplot2” to visualize the differences between the two subtypes. To conduct GO and KEGG analyses, the R package “clusterProfiler” was utilized. CIBERSORT was employed to analyze immune infiltrates.

### Construction and validation of the prognostic risk score

The prognostic risk score was calculated for each sample by multiplying the expression score of the four selected genes by their LASSO coefficients. To assess the prognostic value and the independent prognostic ability of the risk score, Kaplan–Meier (KM) analysis and univariate and multivariate analyses were performed. A nomogram model that included the risk score and the disease stage for patient prognostic prediction at 1-, 3-, and 5-year intervals was created to make it more convenient for clinical use. Calibration and receiver operating characteristic (ROC) and decision curve analysis (DCA) curves were utilized to estimate the calibration, accuracy, and clinical value of the nomogram model.

### Functional and mutational analyses

To calculate the enrichment score for the gene signatures in “msigdb.v7.0.symbols.gmt” and to evaluate variations among subtypes, the GSVA package was employed to perform GSVA. Wilcoxon’s rank-sum test was then utilized to assess variations among subtypes. In order to explore the association of the risk score with mutation features, the somatic mutation data of the 374 HCC samples were retrieved from the TCGA database. The GenVisR algorithm (a package from R Bioconductor) was used to generate a mutation landscape waterfall plot comparing the low and high-risk score groups. The GSEA software was also utilized to perform GSEA.

### Association between the risk score and several mutation and immune indices

MSI, which is a marker of malignancy, was calculated for each sample from the TCGA-LIHC cohort using the R package “PreMSIm.” The mRNA expression-based stemness index (mRNAsi), the DNA methylation-based index (mDNAsi), and the epigenetically regulated mDNAsi and mRNAsi (EREG-mDNAsi and EREG-mRNAsi, respectively), which have been previously identified as stem-like indices and predictors of tumor prognosis, were also calculated to explore the differences among the low- and high risk groups. To compare chromosomal instability, the homologous recombination deficiency (HRD) scores were also determined for low- and high risk groups using the Wilcoxon test, as HRD scores are considered powerful biomarkers for specific cancers. The GSE104580 cohort was obtained from the Gene Expression Omnibus (GEO) database, which includes 147 HCC patients with data on TACE treatment, and was normalized as described above to explore the predictive potential of ICRPI. The Wilcoxon test was used to measure the differences between the low- and high risk groups. A *p*-value <0.5 was considered significant.

### Association between the risk score and immune-related features

The “ESTIMATE” R package was utilized to calculate the immune score, stromal score, and tumor purity based on data from TCGA in order to investigate the potential function of the risk score in HCC and to provide an immune landscape for the disease. In addition, the TIDE method was employed to evaluate the response to immunotherapy and to quantify the TIDE scores for the HCC samples obtained from the TCGA-LIHC cohort. The pan-cancer analysis of immune subtypes by Thorsson et al., (45) which defined six immune-related subtypes, was also taken into account. Furthermore, Cancer Genome Atlas Research Network (46) identified three subtypes of TCGA liver cancer based on unsupervised clustering, with iCluster 1 sharing similar features to proliferative S2 progenitor cells, iCluster 2 having common molecular and pathological features with the non-proliferative type, and iCluster 3 being a new aggressive subtype with chromosomal instability, *TP53* mutations, M2 macrophage infiltration, immune exhaustion, and the worst prognosis. To explore the differences between the immune and molecular subtypes and the risk score, the Wilcoxon test was performed, with *p*-values <0.05 considered statistically significant.

### Drug sensitivity analysis

The predictive value of the risk score in chemotherapy response was evaluated using the “pRRophetic” package to predict the response of HCC to conventional chemotherapeutic drugs based on the Genomics of Drug Sensitivity in Cancer (GDSC) database. Patients were classified into a high- and a low-risk group based on the median risk score, and statistical significance was determined by employing the Wilcoxon rank-sum test with a significance level of *p* < 0.05.

### Cell culture and transfection

HepG2 cells were cultured in Dulbecco’s modified Eagle’s medium (DMEM) supplemented with 10% fetal bovine serum (FBS) under controlled conditions of 5% CO_2_ and 37°C. To knock down the expression of *TMCC1*, Lipofectamine 2000 was used to transfect cells with specific small interfering RNA (siRNA) sequences and a control sequence. The transfection protocol was strictly followed according to the manufacturer’s instructions.

### Quantitative real-time PCR

To measure the mRNA expression levels of the target genes, RNA was extracted from HepG2 cells using the TRIzol reagent. RNA purity and concentration were determined by spectrophotometry. The SYBR Green PCR Master Mix was used for real-time PCR, with cycling conditions of 95°C for 3 min, followed by 40 cycles of 95°C for 15 s and 60°C for 30 s. The relative mRNA expression levels were calculated using the 2^−ΔΔCt^ method, normalized to the expression of glyceraldehyde-3-phosphate dehydrogenase (GAPDH).

### Western blotting

Protein was extracted from cells using a lysis buffer containing protease and phosphatase inhibitors, and the protein concentrations were determined using a BCA assay kit. Equal amounts of protein were loaded onto SDS-PAGE gels and transferred to PVDF membranes. After blocking in 5% non-fat milk in TBST (Tris-buffered saline with Tween-20), the membranes were incubated with primary antibodies at 4°C overnight, followed by secondary antibodies at room temperature for 1 h. GAPDH was used as an internal control.

### Migration assays

For the Transwell assay, HepG2 cells were harvested and resuspended in a serum-free medium, and 1 × 10^5^ cells were seeded onto the upper chamber of Transwell inserts with a pore size of 8 μm. The lower chamber was filled with a medium containing 10% FBS, which served as a chemoattractant. After incubation for 24 h at 37°C, non-migrating cells were removed from the upper surface of the membrane with cotton swabs, while those that had migrated to the lower surface were fixed, stained, and counted under a microscope. The number of migrated cells was quantified by counting at least three randomly selected fields per membrane.

For the wound healing assay, HepG2 cells were seeded onto six-well plates and allowed to grow until ~90% confluence was achieved. A scratch was then made across the cell monolayer using a sterile pipette tip, generating a uniform gap. The cells were then incubated in a medium containing 2% FBS and monitored by microscopy for 48 h. Images were captured at 0 and 24 h to measure the degree of migration. The width of the scratch was measured using ImageJ software, and the percentage of closure was calculated as the ratio of the remaining gap area at 48 h *vs.* that at 0 h.

### Statistical analysis

Statistical analysis was performed using GraphPad Prism software. Data were presented as the mean ± standard deviation (SD). The normal distribution of the data was assessed using the Shapiro–Wilk test. For the comparison of the two groups, an unpaired Student’s *t*-test was used for normally distributed data, while the Mann–Whitney test was used for non-normally distributed data. A *p*-value of <0.05 was considered statistically significant.

## Results

### Identification of M2 macrophage-related genes in hepatocellular carcinoma using CIBERSORT and weighted gene co-expression network analysis

**Figure 1:**
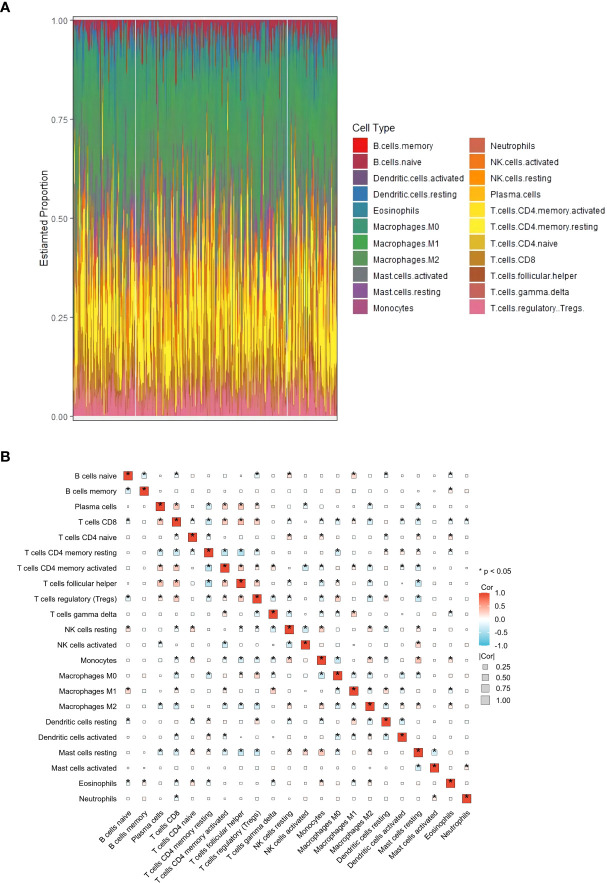
Analysis of immune infiltration in hepatocellular carcinoma (HCC). **(A)** Distribution of immune cell infiltration in each HCC sample using CIBERSORT based on The Cancer Genome Atlas Liver Hepatocellular Carcinoma (TCGA-LIHC) cohort. **(B)** Correlation heat map of 22 immune cell subtypes. *p<0.05.

**Figure 2:**
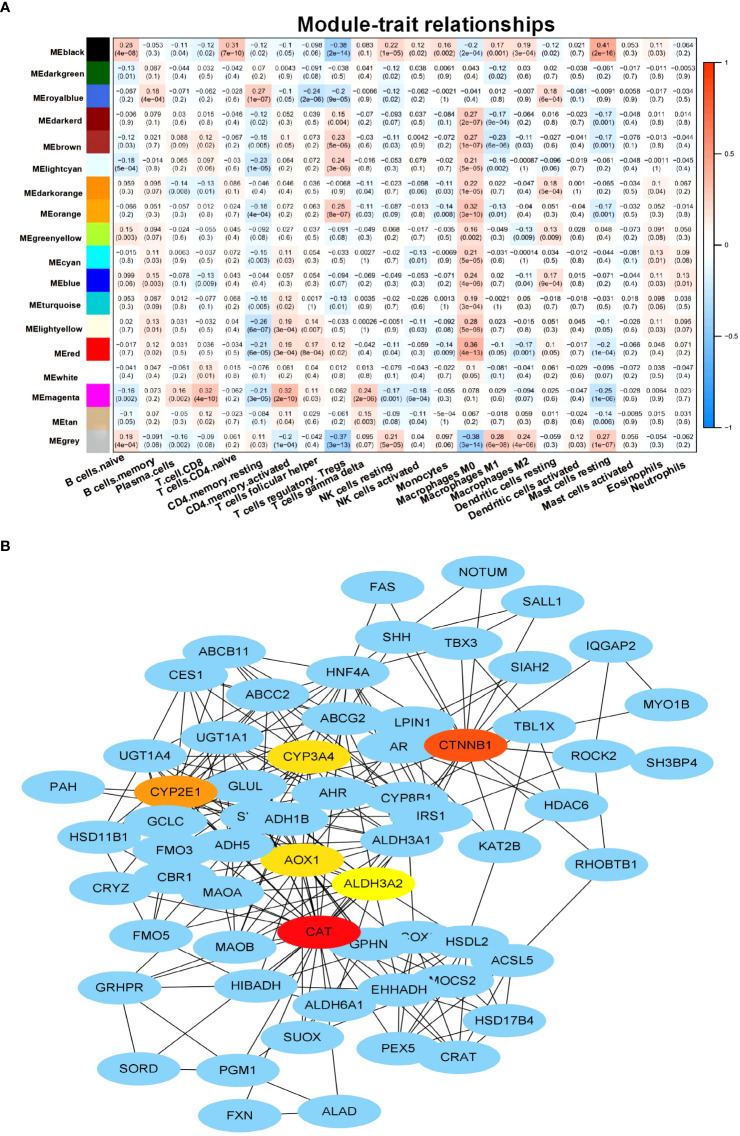
Identification of M2 macrophage-related genes. **(A)** Heat map displaying the correlation between the co-expression modules and immune cells. **(B)** Protein–protein interaction (PPI) networks of the genes in the MEblack model.

This study aimed to identify genes related to M2 macrophages in HCC. To achieve this, CTBERSORT was first used to calculate and demonstrate the immune infiltration of 374 HCC samples in the TCGA-LIHC cohort (**Figure 1A** and **Supplementary Table S1**). Correlation analysis revealed a positive correlation of M2 macrophages with resting natural killer (NK) cells, monocytes, and activated dendritic cells, but a negative correlation with CD8 T cells, activated CD4 memory T cells, T follicular helper (Tfh) cells, and regulatory T cells (Tregs) (**Figure 1B**). Subsequently, 17 co-expression modules within the HCC expression profile were identified using WGCNA, with the MEblack module being closely related to M2 macrophages (*p* = 3e−04) (**Figure 2A** and **Supplementary Figure S1**). Furthermore, GO and KEGG analyses showed that the 158 genes in the MEblack module were mainly enriched in small molecule catabolic processes, fatty acid metabolic processes, and pathways related to drug metabolism (including cytochrome P450) and chemical carcinogenesis (**Supplementary Figure S2**). Finally, a PPI analysis of these genes was performed, which identified six hub genes, namely, *CTNNB1*, *CAT*, *CYP2E1*, *CYP3A4*, *AOX1*, and *ALD3A2* (**Figure 2B** and **Supplementary Table S2**).

### Identification and characterization of the M2 macrophage-related subtypes in hepatocellular carcinoma

**Figure 3:**
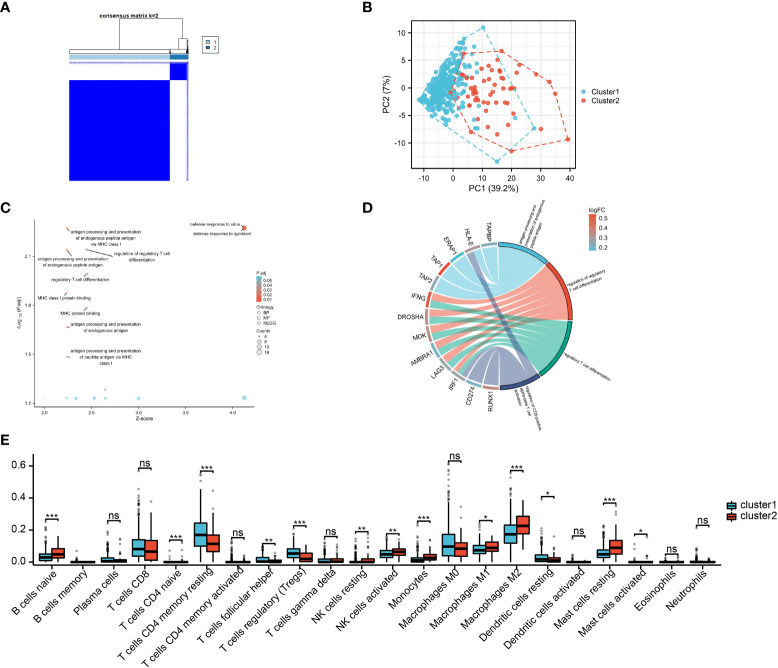
M2 macrophage-related subtypes in hepatocellular carcinoma (HCC). **(A)** Consensus clustering of the 374 HCC samples based on the M2 macrophage-related genes. **(B)** Principal component analysis (PCA) shows the distinctions between two subtypes. **(C, D)** Gene Ontology (GO) and Kyoto Encyclopedia of Genes and Genomes (KEGG) analyses of differentially expressed genes (DEGs). **(E)** Infiltration analysis of 22 immune cell subtypes between two subtypes.

Based on the M2 macrophage-related genes, unsupervised consensus clustering grouped the 374 HCC samples into two clusters: M2 macrophage-related cluster 1 (*n* = 316) and cluster 2 (*n* = 58) (**Figure 3A** and **Supplementary Figure S3** and **Table S3**). PCA showed heterogeneity in the two subtypes of HCC (**Figure 3B**). In order to explore differences in the functional characteristics of the two HCC subtypes, differential gene expression analysis was performed (**Supplementary Table S4**), followed by GO and KEGG analyses to investigate their functional differences. The results revealed that immune-related functions and pathways such as “response to interferon-gamma,” “regulation of CD8-positive, alpha-beta T-cell activation,” and “antigen processing and presentation” were enriched (**Figures 3C, D** and **Supplementary Table S5**). This supported the results of WGCNA showing that the genes in the MEblack module were closely linked to tumor immunity. Moreover, immune infiltration analysis indicated that cluster 2 exhibited higher M2 macrophage infiltration, naive B cells, and resting mast cells, but showed lower Tregs and Tfh cell infiltration (**Figure 3E**).

### Prognostic role of the M2 macrophage-related genes in hepatocellular carcinoma

**Figure 4:**
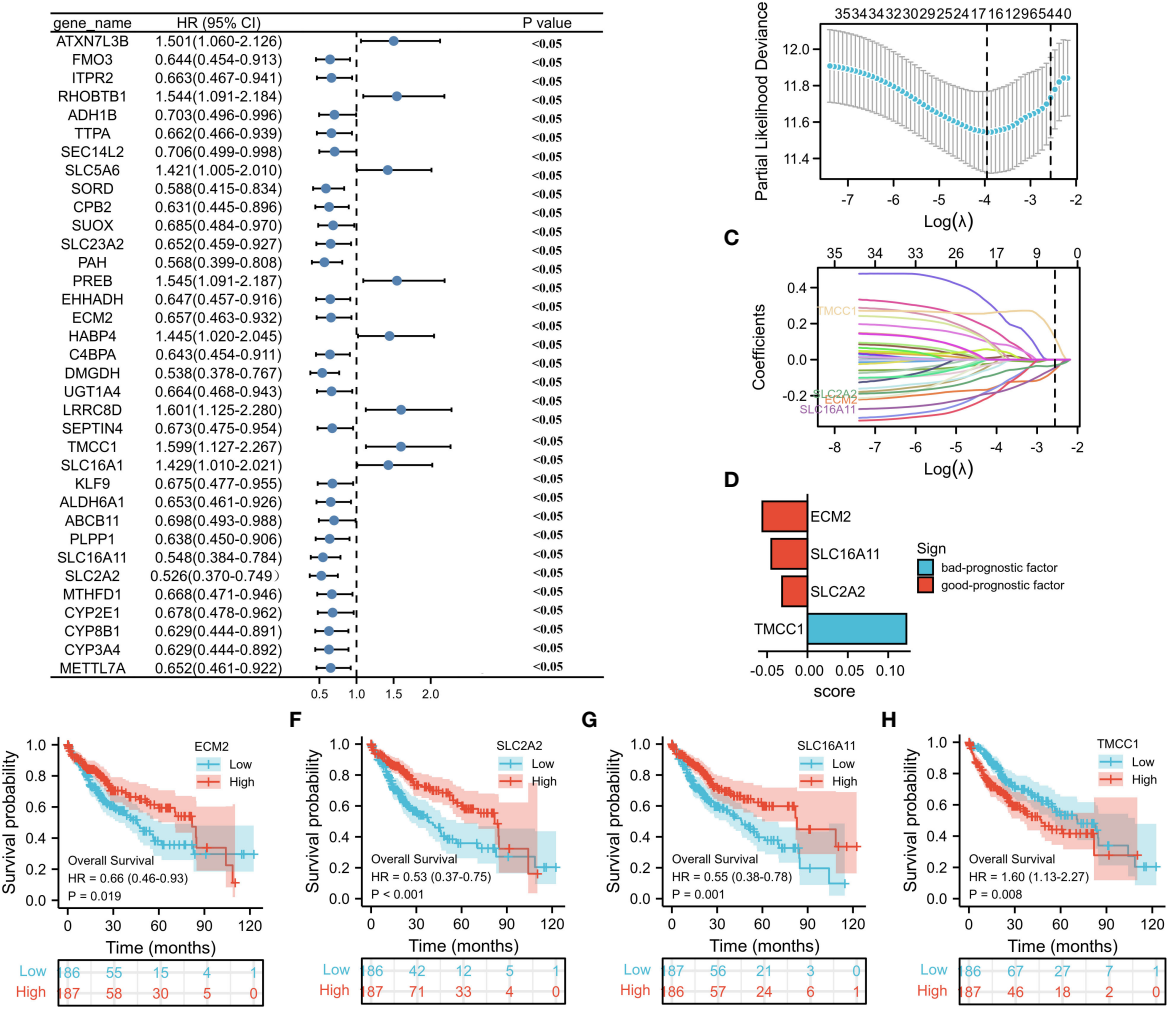
Construction of a prognostic model. **(A)** Forest plot displays the prognostic genes with *p* < 0.05 (*n* = 35) on univariate analysis. **(B, C)** LASSO (least absolute shrinkage selector operator) model for 35 prognostic genes with *p* < 0.05. **(D)** LASSO coefficients of the four selected genes for construction of the prognostic model. **(E–H)** Survival analysis of *ECM2*, *SLC2A2*, *SLC16A11*, and *TMCC1*.

To investigate the prognostic value of the M2 macrophage-related genes in HCC, univariate analysis was performed, which identified 35 genes associated with prognosis (**Figure 4A**). Subsequently, LASSO regression analysis was conducted on these 35 genes. A panel of four genes (i.e., *ECM2*, *SLC16A11*, *SLC2A2*, and *TMCC1*) was ultimately selected to construct a prognostic risk score (**Figures 4B, C** and **Supplementary Table S6**). The risk score for each sample was calculated by multiplying the expression level of each gene with its corresponding risk coefficient (**Figure 4D**). Using the median value of the four selected genes, HCC patients were classified into a high- and a low-expression group, and survival analysis was performed. As illustrated in **Figures 4E–H**, high expression of *TMCC1* and low expression of *ECM2*, *SLC16A11*, and *SLC2A2* were associated with poor prognosis of patients with HCC.

### Prognostic value of the risk score in hepatocellular carcinoma

**Figure 5:**
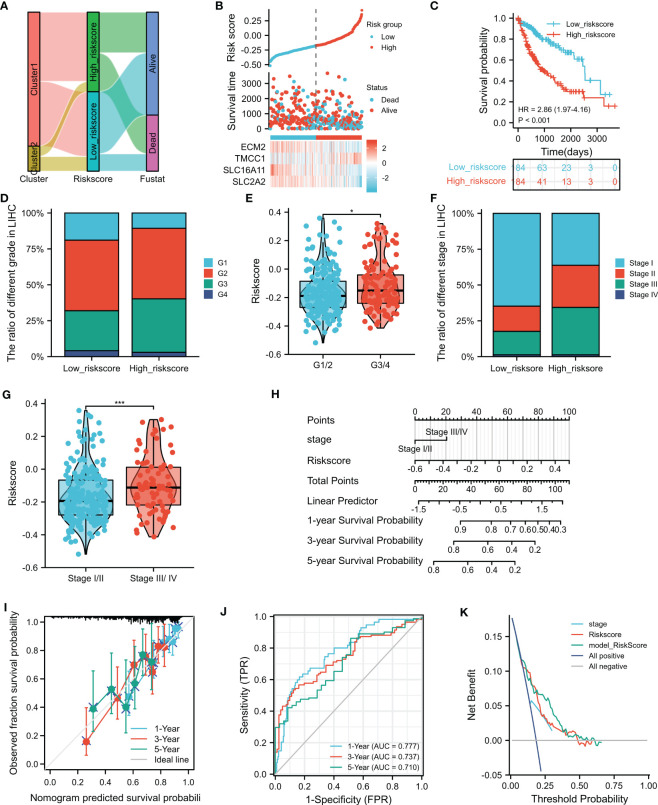
Clinical value of the risk score. **(A)** The ggalluvial diagram displays the relationships among clusters and the risk scores and survival status. **(B)** Distribution of the risk scores, survival status, and the expression of four selected genes in the risk score groups. **(C)** Prognostic performance of the risk score. **(D–G)** Relationship of the risk sores with grades and stages. **(H)** Nomogram for 1-, 3-, and 5-year overall survival (OS) prediction. **(I)** Calibration plot for the consistency test between the predicted and actual observations at 1-, 3-, and 5-year OS. **(J)** Receiver operating characteristics (ROC) showing the performance of the nomogram at 1-, 3-, and 5-year OS. **(K)** Decision curve analysis (DCA) evaluates the clinical effectiveness of the nomogram, risk score, and stage. *p<0.05, ***p<0.001.

To investigate the relationship between the M2 macrophage-related clusters, risk score groups, and survival status, a ggalluvial diagram was used, which depicted that patients in cluster 2 tended to be assigned to the low risk score group, with 78% (144/184) of patients alive in this group (**Figure 5A** and **Supplementary Table S7**). Moreover, patients in the high-risk group exhibited worse survival than those in the low-risk group (**Figures 5B, C**). The associations between the risk score and the clinicopathological features were also explored, which showed a close correlation between advanced HCC grade/stage and high risk score (**Figures 4D–G**). Furthermore, univariate and multivariate analyses showed that the risk score and the disease stage were significantly independent prognostic factors (**Table 1**). These findings were consistent with the validation datasets from the ICGC (**Supplementary Figure S4**). To predict overall survival (OS) at 1, 3, and 5 years, a nomogram based on the risk score and stage was created (**Figure 5H**), which was calibrated by the calibration plot (**Figure 5I**). Moreover, the nomogram had a higher clinical validity than the risk score and stage alone, as indicated by DCA. In summary, the risk score was not only associated with the clinicopathological features but also served as a robust prognostic marker in HCC.

**Table 1:** Univariate and multivariate Cox regression analyses of the clinical features and risk scores.

Characteristics	Total (*N*)	Univariate analysis		Multivariate analysis	
		Hazard ratio (95% CI)	*p*-value	Hazard ratio (95% CI)	*p*-value
Age (years)	343	1.012 (0.998–1.026)	0.102		
Sex	343				
Women	110	Reference			
Men	233	0.841 (0.585–1.209)	0.350		
Grade	338				
G1/2	216	Reference			
G3/4	122	1.150 (0.795–1.664)	0.457		
Stage	319				
Stage I/II	236	Reference			
Stage III/IV	83	2.375 (1.629–3.460)	**<0.001**	2.012 (1.373–2.948)	**<0.001**
Risk score	343	21.517 (8.323–55.627)	**<0.001**	22.159 (7.411–66.251)	**<0.001**

### Comparison of tumor-related features in the different risk score groups

**Figure 6:**
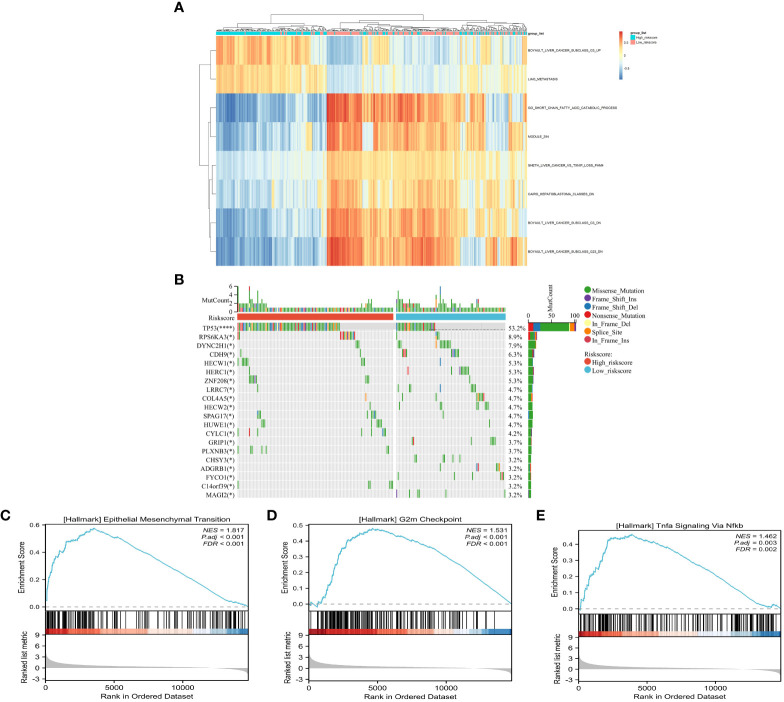
Comparison of the tumor-related features between the high- and low-risk groups. **(A)** Signaling pathway activity between risk groups using gene set variation analysis (GSVA). **(B)** Waterfall plot of the somatic mutations between the low- and high risk score groups. **(C–E)** Identification of the high risk score related pathways using gene set enrichment analysis (GSEA).

The results of the GSVA revealed that “BOYAULT_LIVER_CANCER_SUBCLASS_G3_UP” and “LIAO_METASTASIS” were significantly higher in the high risk score group, while “GO_SHORT_CHAIN_ACID_CATABOLIC_PROCESS” was significantly higher in the low risk score group (**Figure 6A** and **Supplementary Table S8**). Notably, the G3 subtype is typically characterized by mutation of *TP53* and the overexpression of cell cycle genes (22). These findings suggest that patients with HCC in the high risk score group may have a higher capacity for proliferation and invasion. Furthermore, differences in gene mutation in the different risk score groups were observed, and it was found that the mutation rate of *TP53*, as well as that of *RPS6AKA3*, *HECW1*, and *ZNF208*, was significantly higher in the high risk score group, confirming the accuracy of GSVA (**Figure 6B**). In addition, GSEA was utilized for functional enrichment analysis, which showed that “TNFA SIGNALING *via* NFKB,” “EPITHELIAL_MESENCHYMAL_TRANSITION,” and “INFLAMMATORY_RESPONSE” were significantly enriched in the high-risk group, suggesting that, apart from high proliferation and invasion capacity, the immune inflammatory environment may also play a critical role in the high risk score group (**Figures 6C–E** and **Supplementary Table S9**).

### Correlation between genomic features and the risk score

**Figure 7:**
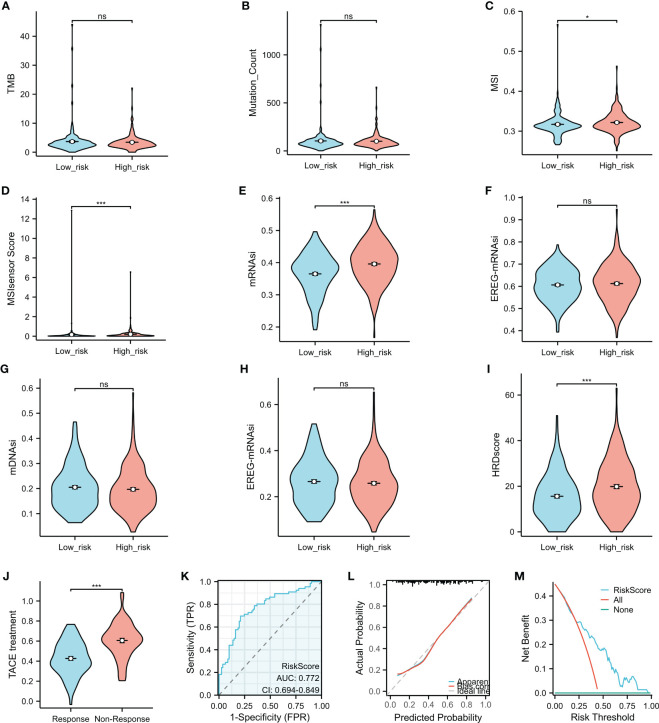
Comparison of the characteristics and transcatheter arterial chemoembolization (TACE) response in the high- and low-risk groups. **(A–I)** The tumor mutation burden (TMB) **(A)**, the Mutation_Count **(B)**, microsatellite instability (MSI) **(C)**, the MSI sensor score **(D)**, mRNAsi **(E)**, EREG-mRNAsi **(F)**, mDNAsi **(G)**, EREG-mDNAsi **(H)**, and the HRD scores **(I)** were compared between the high- and low-risk groups. **(J)** Association between the risk score and TACE response. **(K–M)** The predictive role of the risk score in the response to TACE was assessed using the operating characteristic (ROC) curve **(K)**, calibration plot **(L)**, and decision curve analysis (DCA) **(M)**. *p < 0.05, ***p < 0.001. *ns*, no significance.

Higher tumor mutation burden (TMB) and somatic mutation rates are associated with increased anticancer immunity and greater potential for benefiting from programmed cell death 1/programmed cell death ligand 1 (PD-1/PD-L1) immune checkpoint inhibitors (ICIs). However, there was no significant difference in the TMB or the number of mutated genes (**Figures 7A, B**) between the high- and low-risk groups. MSI, identified as a significant biomarker for various cancer types, was higher in HCC patients in the high-risk group than in those in the low-risk group, as indicated by the MSI and MSI sensor scores (**Figures 7C, D**). The mRNAsi, a novel predictive factor associated with stemness and tumor prognosis, was also higher in the high-risk HCC subgroup compared to the low-risk group (**Figure 7E**). However, there was no statistically significant difference in the EREG-mRNAsi, mDNAsi, or EREG-mDNAsi results (**Figures 7F–H**) between the two groups. The HRD score, representing genome scars and chromosomal instability caused by DNA repair defects, was significantly lower in the low-risk than the high-risk group (**Figure 7I**).

### Risk score as a promising biomarker for predicting response to transcatheter arterial chemoembolization treatment in patients with hepatocellular carcinoma

TACE is a commonly used treatment for unresectable liver cancer, particularly for patients in the advanced stage. Analysis of GSE104580 showed that patients with HCC who did not respond to TACE had significantly higher risk scores than those who responded to the treatment (**Figure 7J**). The risk score showed promising predictive value for HCC response to TACE, with an area under the curve (AUC) of 0.772 (**Figure 7K**), indicating its potential as a biomarker for treatment response prediction. The calibration curves showed that the predictive values of the risk scores agreed with those of actual observations (**Figure 7L**). In addition, the DCA demonstrated that the risk scores improved the net benefits and had a wider range of threshold probability in predicting the HCC responses to TACE (**Figure 7M**) in the training cohort.

### Association between the immune-related features and the risk score

**Figure 8:**
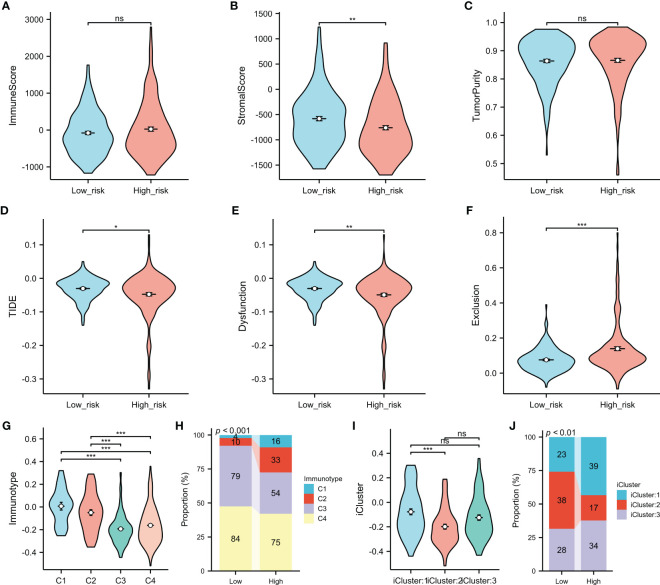
Comparison of the immune-related features between the high- and low-risk groups. **(A–C)** Immune score **(A)**, stromal score **(B)**, and tumor purity **(C)** between the low and high risk score groups were determined using the ESTIMATE algorithm. **(D–F)** Association of the risk score with TIDE **(D)**, dysfunction **(E)**, and exclusion **(F)**. **(G–J)** Association of the risk score with the immune subtypes **(G, H)** and molecular subtypes **(I, J)**. *p < 0.05, **p < 0.01, ***p < 0.001. *ns*, no significance.

Analysis using the ESTIMATE algorithm revealed no significant differences in the immune scores or the tumor purity between the low- and high-risk groups (**Figures 8A, C**); however, the low-risk group had a higher stromal score (**Figure 8B**). Higher TIDE scores indicated a lower likelihood of responding to ICI treatments. In contrast, HCC patients in the high-risk subgroup were more likely to benefit from ICI treatments, as suggested in **Figures 8D–F**. Patients in Cluster 1 and 2 (C1 and C2) had higher overall risk scores compared to those in Cluster 3 and 4 (C3 and C4) (**Figure 8G**), with more HCCs in the high-risk subgroup classified into C1 and C2 compared to the low-risk subgroup (**Figure 8H**). Specifically, 27.5% of HCCs in the high-risk subgroup (C1 = 9.0%, C2 = 18.5%) were classified into C1 and C2 compared to only 7.9% in the low-risk subgroup (C1 = 2.2%, C2 = 5.5%). In addition, HCCs in iCluster 2 had the lowest risk scores (**Figure 8I**), with the proportion bar chart showing that the number of patients in iCluster 2 in the high-risk group was roughly twice that in the low-risk group (38 *vs*. 17) (**Figure 8J**).

### Evaluation of the risk score for predicting sensitivity to chemotherapeutic drugs

**Figure 9:**
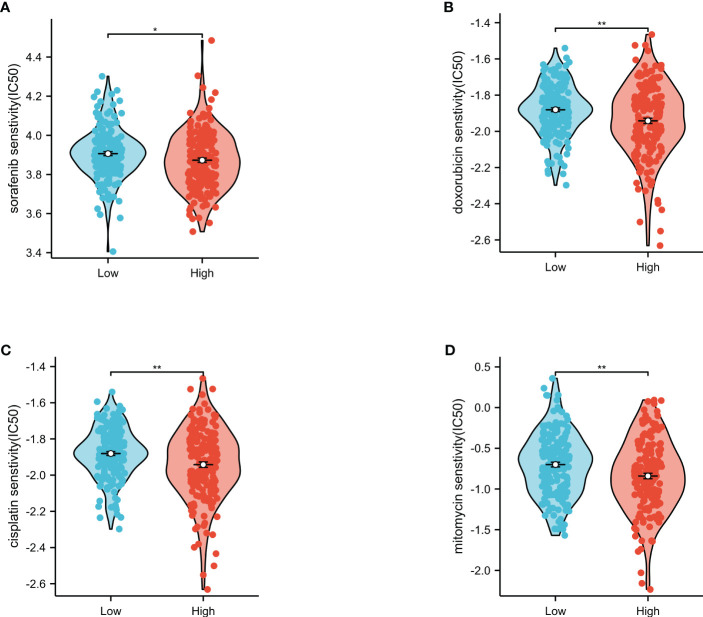
Evaluation of the IC_50_ values of chemotherapeutic drugs in the low- and high-risk groups. Sorafenib **(A)**, doxorubicin **(B)**, cisplatin **(C)**, and mitomycin **(D)** in The Cancer Genome Atlas (TCGA). *p<0.05, **p<0.01.

Using the GDSC database, this study assessed the value of the risk score in predicting the sensitivity of the high- and low-risk groups to chemotherapeutic drugs (**Figure 9**). The results showed that the high-risk group had significantly lower IC_50_ values for all four chemotherapeutic drugs (i.e., sorafenib, doxorubicin, cisplatin, and mitomycin) compared to the low-risk group. These findings suggest that the risk score is a potential predictor for chemotherapy efficacy.

### Verification of risk score-related gene expression

**Figure 10:**
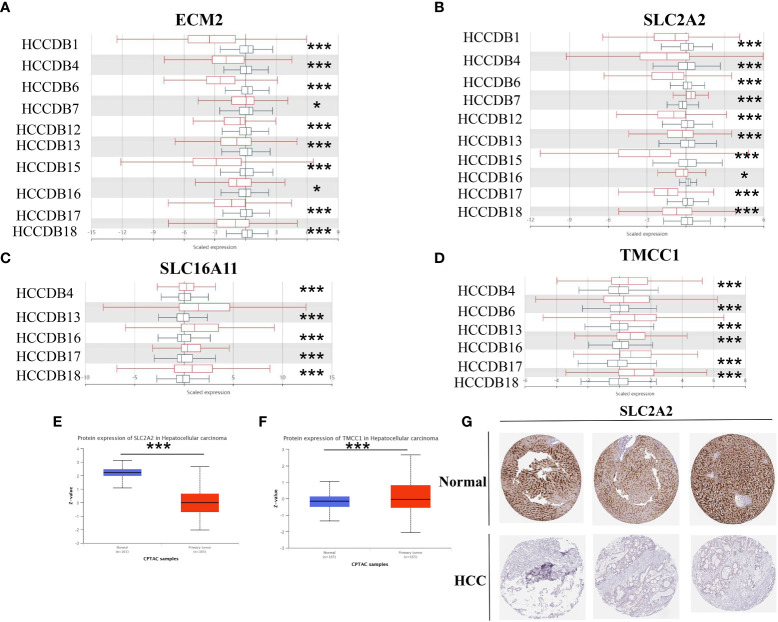
Study of the expression of M2 macrophage-related genes in hepatocellular carcinoma (HCC). **(A–D)** The mRNA expression of *ECM2* **(A)**, *SLC2A2* **(B)**, *SLC16A11* **(C)**, and *TMCC1* **(D)** in the HCCDB database. **(E, F)** Protein expression of *SLC2A2* **(E)** and *TMCC1* **(F)** in HCC based on samples from the Clinical Proteomic Tumor Analysis Consortium (CPTAC). **(G)** Immunohistochemical images of the *SLC2A2* protein in normal and HCC tissues from the Human Protein Atlas (HPA). *p<0.05, ***p<0.001.

Using the HCCDB database, this study investigated the expression of four genes associated with macrophage-related risk scores in HCC and normal liver tissues. *ECM2* and *SLC2A2* showed lower expression trends in HCC tissues, which was validated in 10 different datasets containing both HCC and normal liver tissues (**Figures 10A, B**). Conversely, *SLC16A11* and *TMCC1* showed higher expression in HCC tissues, which was validated in five and six different HCC datasets, respectively (**Figures 10C, D**). At the protein expression level, using HCC samples and liver cancer samples from the Clinical Proteomic Tumor Analysis Consortium (CPTAC), it was further confirmed that *SLC2A2* was lower expressed and *TMCC1* was more highly expressed in HCC tissues (**Figures 10E, F**). Immunohistochemical analysis using images extracted from the Human Protein Atlas (HPA) revealed SLC2A2 to have high staining in normal liver tissues, while no staining was detected in HCC tissues (**Figure 10G**).

### *TMCC1* promotes HepG2 cell migration through the Wnt signaling pathway

**Figure 11:**
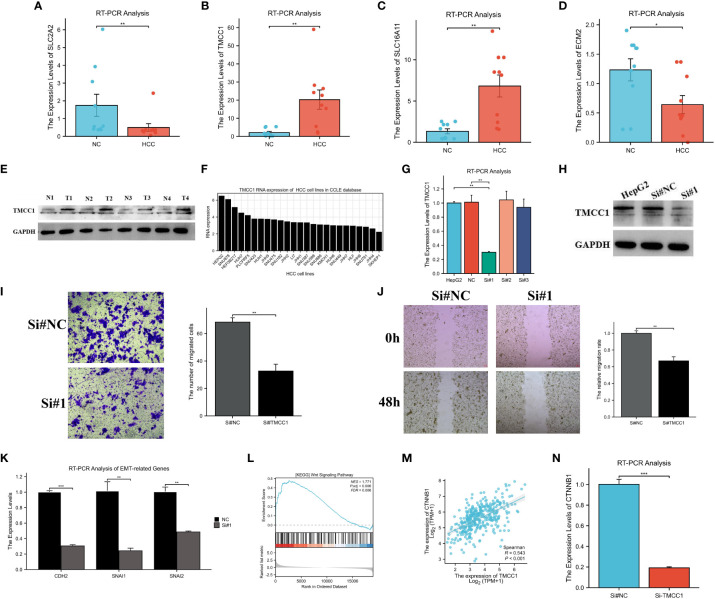
Experimental verification of the results of bioinformatics analysis. **(A–D)** RT-PCR analysis of the mRNA expression of *ECM2* **(A)**, *SLC2A2* **(B)**, *SLC16A11* **(C)**, and *TMCC1* **(D)** in hepatocellular carcinoma (HCC) and normal tissues. **(E)** Protein expression of *TMCC1* in paired HCC tissues. **(F)** Exploration of the RNA levels of *TMCC1* in different liver cancer cell lines using the Cancer Cell Line Encyclopedia (CCLE). **(G, H)** Verification of the efficiency of *TMCC1* knockdown at the RNA **(G)** and protein **(H)** levels. **(I, J)** Transwell **(I)** and wound healing **(J)** assays exploring the effect of *TMCC1* on the migration function of HepG2 cells. **(K)** RT-PCR analysis of the changes in EMT-related gene expression following *TMCC1* manipulation. **(L)** Gene set enrichment analysis (GSEA) of *TMCC1* based on The Cancer Genome Atlas Liver Hepatocellular Carcinoma (TCGA-LIHC) dataset. **(M)** Correlation analysis between *TMCC1* and *CTNNB1*. **(N)** RT-PCR analysis of the effect of *TMCC1* gene knockdown on *CTNNB1*. *p<0.05, **p<0.01, ***p<0.001.

First, the expression of four genes in HCC tissues was validated using RT-PCR. It was found that, compared to adjacent non-cancerous tissues, *TMCC1* and *SLC16A11* were upregulated, while *SLC2A2* and *ECM2* were downregulated, which was consistent with the results of previous analyses (**Figures 11A–D**). Of these four genes, *TMCC1* was highly expressed in HCC and was associated with poor prognosis, but its function in HCC is unknown. Therefore, the role of *TMCC1* in liver cancer cells was investigated. The protein expression of *TMCC1* was found to be higher in HCC tissues compared to adjacent non-cancerous tissues (**Figure 11E**). Regarding the liver cancer cell line, HepG2 exhibited the highest *TMCC1* RNA level in the Cancer Cell Line Encyclopedia (CCLE); thus, HepG2 cells were selected as the cell model with a knocked-down *TMCC1* gene (**Figure 11F**). The knockdown efficiency experiments showed that the knockdown of *TMCC1* using Si#1 effectively reduced its expression at both the RNA and protein levels (**Figures 11G, H**). Therefore, we chose Si#1 as the knockdown sequence for subsequent experiments. Moreover, the knockdown of *TMCC1* significantly inhibited the migration of HepG2 cells, as shown by the Transwell and wound healing assays (**Figures 11I, J**). The knockdown of *TMCC1* also resulted in a significant reduction of the expression of EMT-related genes at the RNA level (**Figure 11K**). GSEA indicated that the Wnt signaling pathway was significantly enriched and that *TMCC1* was positively correlated with *CTNNB1* (**Figures 11L, M**). The PCR experiments revealed that the knockdown of *TMCC1* led to a significant decrease in the expression of *CTNNB1* at the RNA level (**Figure 11N**). In summary, *TMCC1* promotes HepG2 cell migration through the Wnt signaling pathway.

## Discussion

HCC, the major histological type of liver cancer, is a serious health concern with high incidence and mortality (23). Despite advances in the diagnosis and treatment of this disease, the effects have not been satisfactory (24). There is an urgent need for prognostic markers to stratify patients for clinical management.

Macrophage infiltration is a common phenomenon in most solid tumor malignancies. Based on previous cancer research, macrophages can be classified into tumor-suppressing M1 macrophages and tumor-promoting M2 macrophages. Although current studies have found that macrophage polarization is plastic and complex and that the M1/M2 model does not recapitulate the more complex, mixed functional profiles of macrophages *in vivo* in inflamed tissues, extensive studies have shown that the M1 and M2 types have a discordant effect on the evolution and progression of tumors. The M1 tumor-associated macrophage (TAM) phenotype counteracts tumor progression, while M2 TAM promotes tumor growth; moreover, the M2 TAM phenotype is important in growth and metastasis during cancer development (25–27). Therefore, the M1/M2 model of macrophages has certain rationality and applicability in cancer research. However, with regard to the tumor-promoting subtype, the prognostic value of M2 macrophages in HCC remains unclear. In this study, two M2 macrophage-related subtypes were identified and a four-gene prognostic model including *ECM2*, *SLC16A11*, *SLC2A2*, and *TMCC1* was constructed to predict OS in HCC. *SLC2A2* has been reported as a prognostic marker of HCC, with an effect on the alteration of the TME (28). Recently, researchers have constructed a prognostic model using endoplasmic reticulum (ER) stress-related genes (ERSRGs), including *PON1*, *AGR2*, *SSR2*, and *TMCC1*, which could accurately predict the survival outcomes of patients with HCC and also be associated with malignant degree, recurrence rate, late TNM stage, late T stage, and hepatitis B virus (HBV) infection (29).

Type I interferon signals play important roles in priming antitumor CD8 T-cell responses (30). They can enhance the sensitivity of tumor macrophages to interferon-gamma (IFN-γ) through the JAK/STAT1 pathway (31). Furthermore, Tregs could be attracted by TAMs through the release of chemokine CCL22 (32). In KEGG and GO analyses, the “type I interference signaling pathway” and “regulation of regulatory T-cell differentiation” showed significant differences in the two subtypes. Immune infiltration analysis showed that M2 macrophages were significantly higher in cluster 2 than in cluster 1. These results showed that the MEblack module was closely related to tumor immunity, especially macrophages, further confirming the results of WGCNA. Boyault et al. classified liver cancer into subgroups G1–G6 using unsupervised transcriptome analysis, with the G3 subgroup being characteristic of *TP53* mutation and the overexpression of cell cycle-related genes (22). In the GSVA, the G3 subgroup score of the high-risk group was higher. In addition, “LIAO-METASTASIS” was also enriched in the high-risk group. Verifying the results of GSVA, mutation analysis confirmed the high *TP53* mutation in the high risk score group. As a tumor suppressor that regulates cell cycle, apoptosis, and senescence, *TP53* is mutated and inactivated in most tumors, and its inactivation state promotes the occurrence and development of tumors (33–35). In postoperative breast cancer patients, the levels of *TP53* were positively associated with the risk of tumor recurrence (36). In addition, studies have shown that the expression of *TP53* is associated with the aggressive behavior of pituitary tumors (37). Moreover, the liver cancer-related driver genes *RPS6KA3* and *DYNC2H1* and the tumor migration-related gene *CDH9* were also significantly different between the two groups (38). GSEA further confirmed that the risk score was associated with tumor immunity, tumor migration, and proliferation.

As a quantitative index of cancer stem cells (CSCs) and a measure of tumor stem-like features, mRNAsi serves as a predictor that is closely associated with both stemness and tumor prognosis. Higher mRNAsi values have been found to be correlated with stronger tumor stemness and poorer prognosis (39). Our study further supports the importance of mRNAsi by showing its positive association with the risk score, which suggests that mRNAsi is a significant risk factor for the survival of patients with HCC (40). Another crucial biomarker used in therapy decision-making is HRD, which refers to deficiencies in the homologous recombination repair (HRR) mechanisms of DNA repair. A positive HRD status is associated with increased sensitivity to poly(ADP-ribose) polymerase (PARP) inhibitors, further emphasizing the potential value of HRD testing as a predictive biomarker for PARP inhibitor treatment (41). Notably, HRD scores can predict response to chemotherapy in certain cancers, such as triple-negative breast cancer, and are associated with poor survival rates in HCC (42, 43). Lastly, our findings suggest that the risk score could potentially help evaluate the effectiveness of TACE treatments for HCC, indicating that this tool could be utilized to select the most appropriate treatment methods for patients with HCC.

The TIDE score has emerged as a valuable alternative biomarker for predicting response to ICI therapy, where higher TIDE scores indicate a lower likelihood of benefiting from ICI treatment (44). This study posits that patients with high-risk subgroups of HCC in the high risk score group may benefit more from ICI treatment. Our investigation into the immune classification of HCC, C1–C4, demonstrated that patients with higher matrix and immune scores (C1 and C2) had higher risk scores, while subtype 2 (proliferative) had lower risk scores and subtypes 1 (metabolic) and 3 (mixed) had higher risk scores, indicating the potential of the risk score as a tool to differentiate the HCC molecular subtypes. The relationship between the risk score and the sensitivity to four common tumor treatment drugs—sorafenib, doxorubicin, cisplatin, and mitomycin—was then investigated, and it was found that HCC patients in the high-risk group with high risk scores exhibited increased sensitivity to these four drugs, suggesting risk score as a means of predicting HCC patients’ sensitivity to these chemotherapeutic agents.

This study had limitations, which included the lack of external validation using our own data, with the analysis relying solely on public HCC cohorts. In future research, we plan to include patients with HCC to further validate the risk score. In addition, the function of the *TMCC1* gene in HCC was examined in *in vitro* cell experiments; therefore, further validation in animal experiments is required. Moreover, only metastasis was studied, leaving other malignant phenotypes such as tumor cell proliferation, cell cycle, and cell apoptosis unexplored. Finally, while the M1/M2 model was used to classify macrophages in the subsequent bioinformatics analysis and experimental verification, this model may not be applicable in all diseases as it oversimplifies the functional complexity of macrophages. Future experiments should be designed to further understand the complex role of macrophages in the development of HCC.

In summary, this study offers novel insights into enhancing individualized survival estimates and treatment responses, such as TACE, immunotherapy, and drug therapy, through a holistic analysis of M2 macrophages. These findings may have significant implications for the development of personalized and precise immunotherapeutic strategies for patients with HCC in the future.

## Conclusion

This study first screened out 158 M2 macrophage-related genes in HCC. Two M2 macrophage-related subtypes were identified and an M2 macrophage-related prognostic model was constructed. The performance of this predictive model was confirmed using an independent external cohort. This research could promote our understanding of the role of M2 macrophages and provides novel prognostic biomarkers and therapeutic targets in HCC.

## Data availability statement

The datasets presented in this study can be found in online repositories. The names of the repository/repositories and accession number(s) can be found below: TCGA, TCGA-LIHC, dbGaP Study Accession phs000178, and the ICGC-LIRI-JP datasets, retrieved from the HCCDB data portal (http://lifeome.net/database/hccdb).

## Author contributions

HS and JH designed the research. HS, XW, and CZ analyzed the data. HS, XW, and CZ performed the experiment and the statistical analysis. HS, XW, and CZ wrote the paper. JH contributed to revising the manuscript. All authors reviewed the manuscript. All authors contributed to the article and approved the submitted version.
