# Supplementary figures and images for: Construction of an M2 macrophage-related prognostic model in hepatocellular carcinoma
Source: Front Oncol. 2023 Jun 20;13:1170775. doi: 10.3389/fonc.2023.1170775 (PMC10319018; doi:10.3389/fonc.2023.1170775)

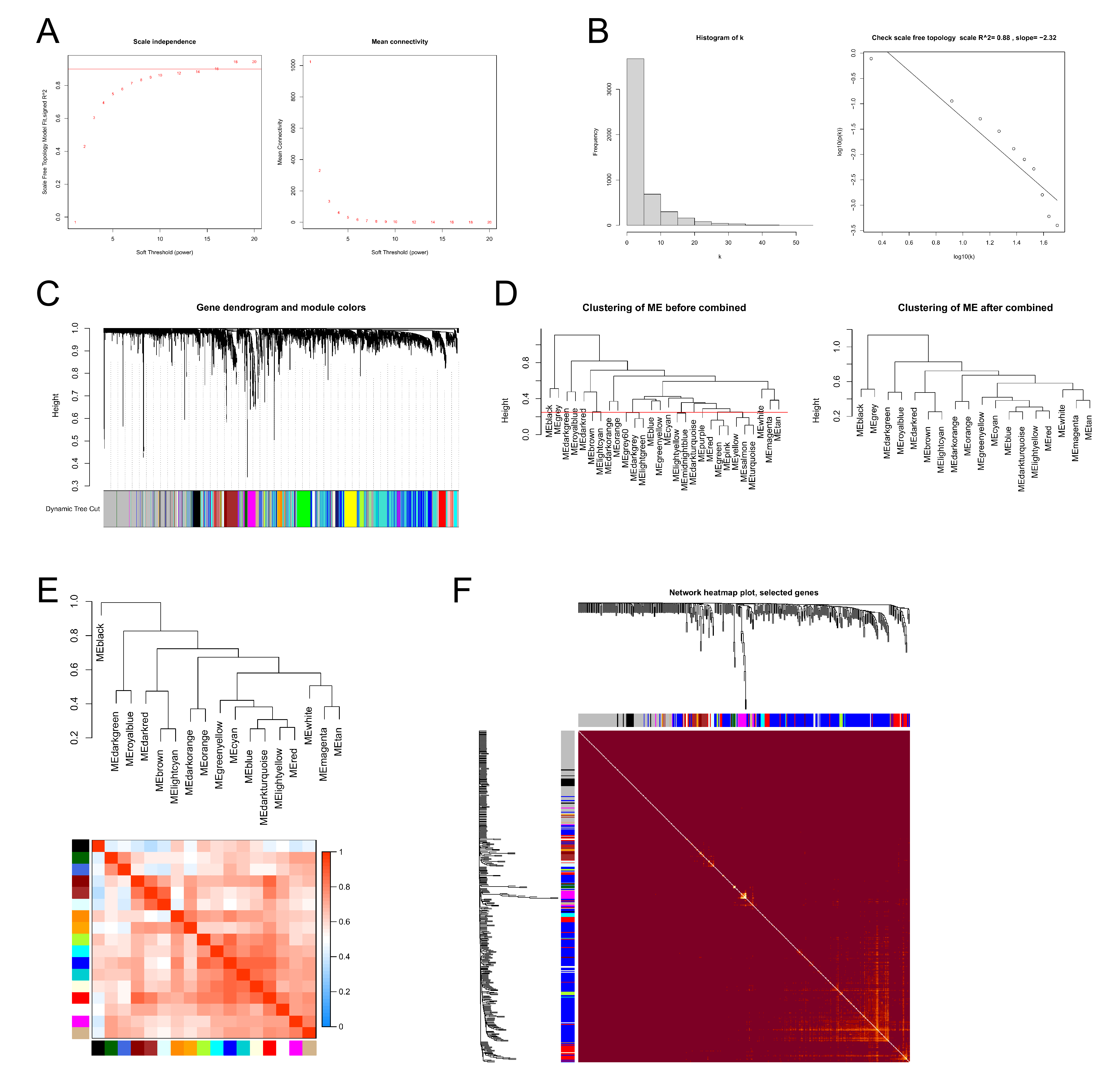

Supplement: Supplementary file 1 [file Image_1.tiff]

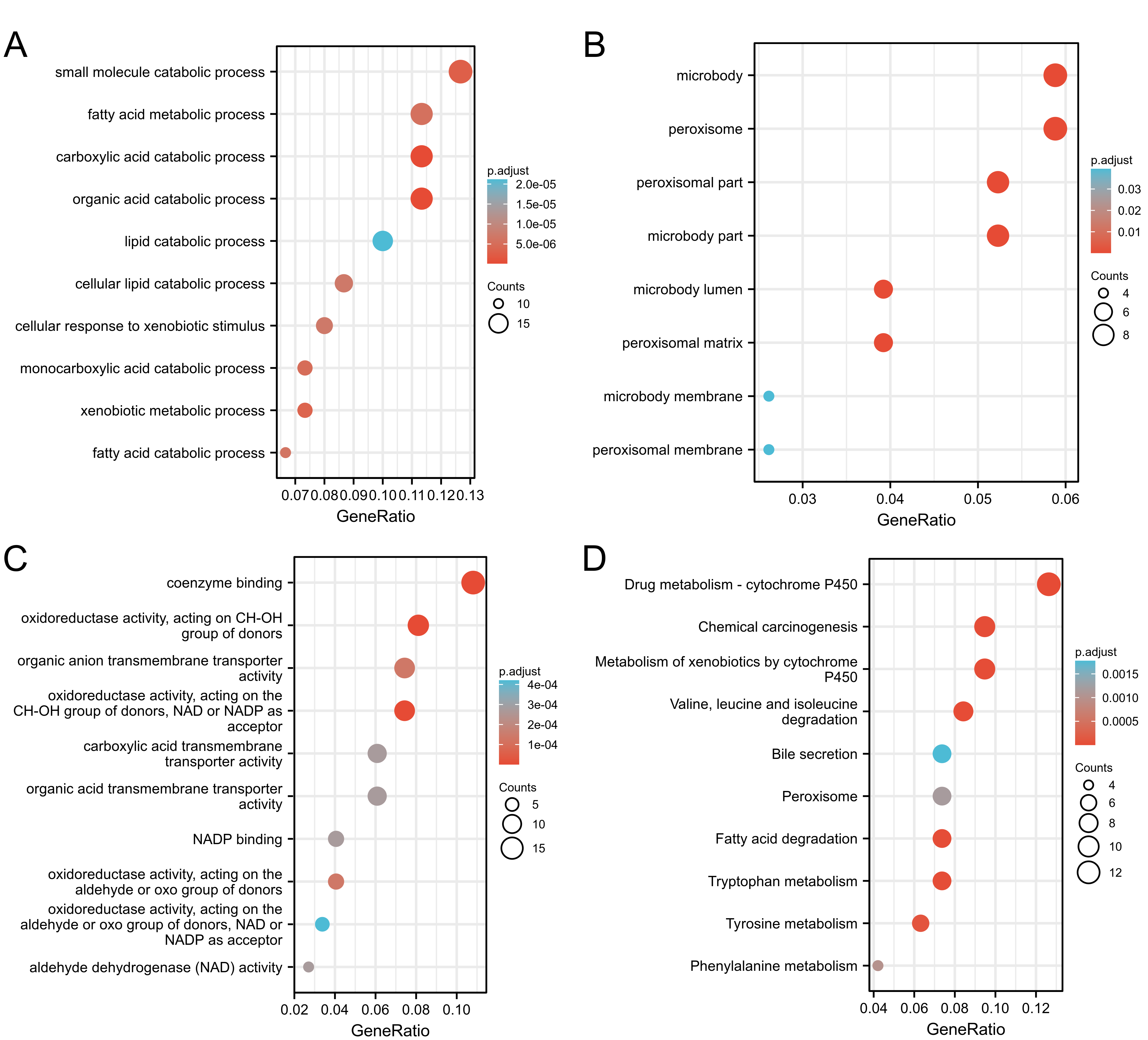

Supplement: Supplementary file 2 [file Image_2.tiff]

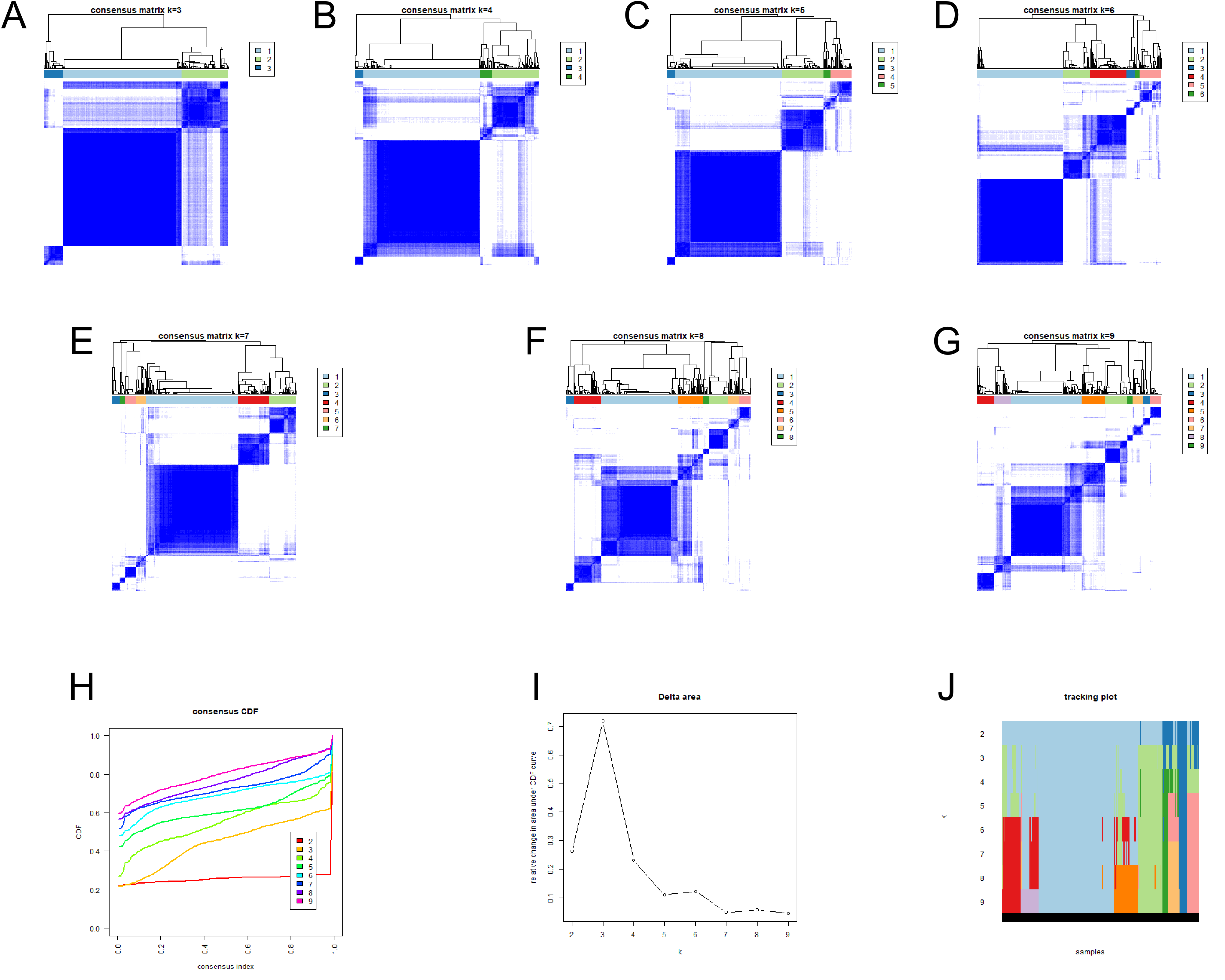

Supplement: Supplementary file 3 [file Image_3.tiff]

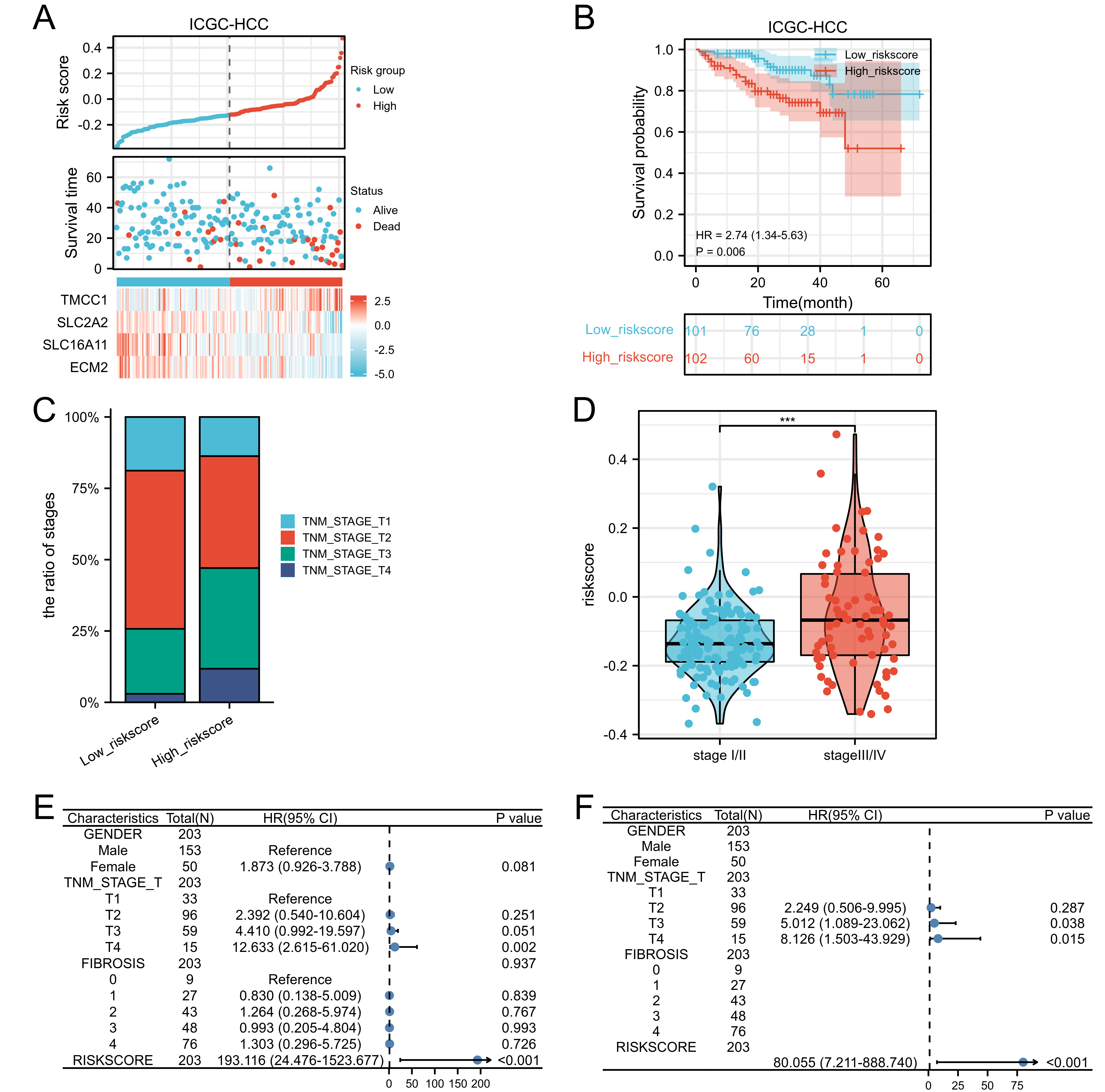

Supplement: Supplementary file 4 [file Image_4.tiff]
